# TAO-DFT Study on the Electronic Properties of Diamond-Shaped Graphene Nanoflakes

**DOI:** 10.3390/nano10061236

**Published:** 2020-06-25

**Authors:** Hong-Jui Huang, Sonai Seenithurai, Jeng-Da Chai

**Affiliations:** 1 Department of Physics, National Taiwan University, Taipei 10617, Taiwan; r07222035@ntu.edu.tw (H.-J.H.); seenithurai@gmail.com (S.S.); 2 Center for Theoretical Physics and Center for Quantum Science and Engineering, National Taiwan University, Taipei 10617, Taiwan

**Keywords:** TAO-DFT, electronic properties, graphene nanoflakes, radical nature, strong static correlation

## Abstract

At the nanoscale, it has been rather troublesome to properly explore the properties associated with electronic systems exhibiting a radical nature using traditional electronic structure methods. Graphene nanoflakes, which are graphene nanostructures of different shapes and sizes, are typical examples. Recently, TAO-DFT (i.e., thermally-assisted-occupation density functional theory) has been formulated to tackle such challenging problems. As a result, we adopt TAO-DFT to explore the electronic properties associated with diamond-shaped graphene nanoflakes with *n* = 2–15 benzenoid rings fused together at each side, designated as *n*-pyrenes (as they could be expanded from pyrene). For all the *n* values considered, *n*-pyrenes are ground-state singlets. With increasing the size of *n*-pyrene, the singlet-triplet energy gap, vertical ionization potential, and fundamental gap monotonically decrease, while the vertical electron affinity and symmetrized von Neumann entropy (which is a quantitative measure of radical nature) monotonically increase. When *n* increases, there is a smooth transition from the nonradical character of the smaller *n*-pyrenes to the increasing polyradical nature of the larger *n*-pyrenes. Furthermore, the latter is shown to be related to the increasing concentration of active orbitals on the zigzag edges of the larger *n*-pyrenes.

## 1. Introduction

Graphene, a wonder material consisting of a hexagonal arrangement of carbon atoms in a plane, has revolutionized science and technology over the past few decades [1,2]. The covalent bonds formed from the $sp^{2}$-hybridized carbon atoms are responsible for the structure of graphene. Besides, graphene exhibits the Dirac cone that is suitable for electronics applications. Graphene also has excellent electronic, mechanical, thermal, and optical properties. However, graphene has been found to be a zero-gap semiconductor or semimetal. Therefore, in order to operate graphene as a semiconductor material in electronic devices, it is essential to adopt some scenarios to introduce a band gap in graphene, such as defect formation, doping, functionalization, and many others.

Among them, one scenario to tune the properties of graphene is to cut an infinite graphene sheet into nano-sized graphene fragments, such as graphene nanoflakes (GNFs). GNFs can be classified based on their shapes (e.g., rectangular [3,4,5,6,7], disk-like [8], hexagon-shaped [9,10,11,12,13], triangle-shaped [11,14,15], bowtie-shaped [16], and many other shaped [17,18,19,20,21] GNFs) and their edges (e.g., zigzag and/or armchair [11] GNFs). In particular, zigzag GNFs could possess room-temperature magnetism as well as adjustable energy gaps [13]. Also, the optical and electronic properties associated with graphene nanoribbons (GNRs) (i.e., rectangular GNFs) could be tuned by using uniaxial strain [7]. GNRs (i.e., rectangular GNFs) have large hydrogen storage capacity [4] and interesting magnetic properties [3]. Besides, triangle-shaped GNFs linked by carbon chains can have intrinsic ferromagnetism [19]. In addition, topological frustration in GNFs can yield large net spin [17]. Moreover, the edge states and termination of edges using different atoms lead to interesting properties.

The structural versatility of GNFs gives rise to an interesting avenue for the exploration of the properties of GNFs with different shapes, edges, sizes, lengths/breadths, edge-termination, pores, and so forth [11,14,21,22]. The interesting electronic and magnetic properties and tunability of properties make these GNFs potential candidates for electronics, spintronics [16], nanoscale molecular logic gates [23], and so on. Besides, GNFs can be used as the building blocks of novel nanomaterials [19]. Large-spin GNFs can be used in nanoscale spintronics devices [17]. In addition, graphene quantum dots are potential candidates for spin memory, transistors, and solid-state qubits in quantum computer applications [24,25].

**Figure 1:**
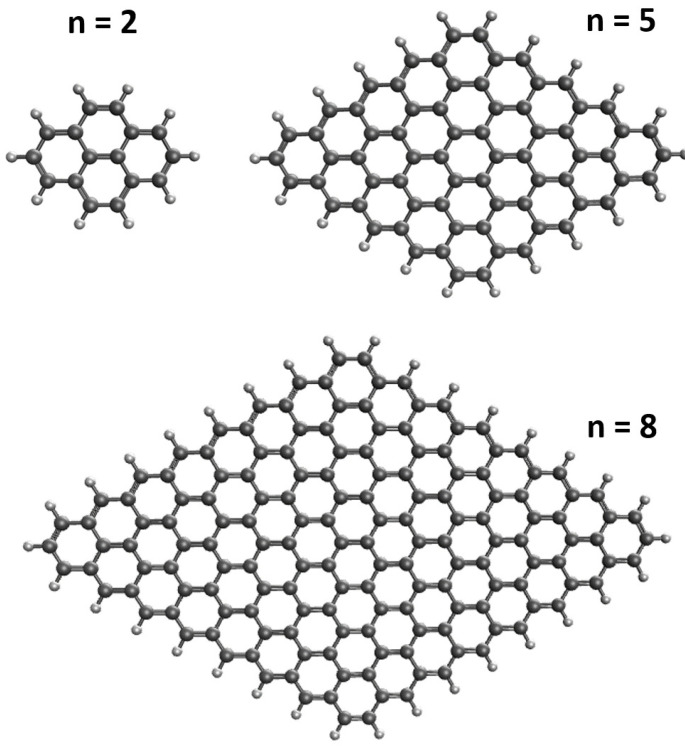
Structure of *n*-pyrene, which contains *n* benzenoid rings fused together at each side.

Despite flourishing activities in studying the properties and applications of different GNFs, there are only scarce studies on diamond-shaped GNFs (i.e., rhombic GNFs). A diamond-shaped GNF can be viewed as two interconnected triangle-shaped GNFs [15]. It is also worth mentioning that triangulene, which can be viewed as a small triangle-shaped GNF, has been recently synthesized by Pavliček et al. [26]. Since the larger triangle-shaped GNFs has been found to possess pronounced polyradical character [15], it may be of interest to investigate whether the polyradical character can persist for the closely related diamond-shaped GNFs. Therefore, in this work, we explore the electronic properties associated with diamond-shaped GNFs with *n* benzenoid rings fused together at each side (designated as *n*-pyrenes), which could be expanded from pyrene (see Figure 1). Note that *n*-pyrene (with the chemical formula C${}_{2n^{2}+4n}$H${}_{4n+2}$), which contains $N=12n^{2}+28n+2$ electrons, belongs to the class of PAHs (i.e., polycyclic aromatic hydrocarbons). Particularly, 2-pyrene (i.e., pyrene), a naturally occurring molecule in interstellar space [27], has been synthesized by Förster and Kasper [28]. However, owing to the difficulties in synthesizing the larger *n*-pyrenes, there are few experimental studies of the larger *n*-pyrenes.

Consequently, theoretical studies can be complementary, and could offer useful information on the properties of *n*-pyrenes (i.e., diamond-shaped GNFs of different sizes). Because of the pronounced radical character found in the closely related triangle-shaped GNFs [15] and because of their low dimensionality [29], *n*-pyrenes are expected to possess radical character, and hence they can be troublesome electronic systems for common computational approaches. For systems possessing radical character, KS-DFT (i.e., Kohn-Sham density functional theory) [30] with the commonly employed XC (i.e., exchange-correlation) energy functionals is prone to predict unreliable results [31,32]. In order to explore the properties associated with such electronic systems, one generally resorts to multi-reference (MR) electronic structure methods, including the complete-active-space self-consistent-field (CASSCF) and related methods [33,34,35,36,37,38,39]. While these MR electronic structure methods are reliably accurate, they are computationally expensive, and hence intractable for large electronic systems. Therefore, efficient methods are necessary for performing the electronic structure calculations on the larger *n*-pyrenes.

Recently, TAO-DFT (i.e., thermally-assisted-occupation density functional theory) [40] has been developed for studying the electronic properties associated with nanosystems exhibiting radical character. In strong contrast to KS-DFT, we emphasize that TAO-DFT is a DFT (i.e., density functional theory) employing fractional orbital occupation numbers (given by the Fermi-Dirac distribution function with the fictitious temperature θ). In TAO-DFT, an entropy contribution component, which depends on the θ value as well as the orbital occupation numbers, is capable of providing an adequate description for strong static correlation even when the very simple local density approximation (LDA) XC energy functional is employed. Relatively complicated semilocal [41], common hybrid [42] or long-range corrected hybrid [42,43] XC energy functionals could be used for performing the TAO-DFT calculations as well. Note that TAO-DFT, which is similar to KS-DFT in computational complexity, reduces to KS-DFT for electronic systems exhibiting nonradical character. Besides, to improve the overall accuracy of TAO-DFT for diverse applications, an approach that determines the self-consistent value of θ in TAO-DFT has been recently formulated [44]. Also, to comment on its applicability, TAO-DFT has been widely adopted for electronic structure calculations [5,12,15,45,46,47,48,49,50,51,52,53], hydrogen storage applications [46,48,49], and vibrational analysis [54], especially for nanosystems possessing radical character. Furthermore, in several recent investigations [5,37,39,40,42,45], the occupation numbers of orbitals from TAO-DFT have been shown to be close to the occupation numbers of natural orbitals obtained with the variational two-electron reduced-density-matrix-driven CASSCF (v2RDM-CASSCF) method, which is an accurate MR electronic structure method, leading to a qualitatively similar tendency in describing the radical nature associated with various PAHs.

In particular, TAO-DFT has been employed to explore the electronic properties associated with zigzag GNFs of different shapes and sizes, including zigzag GNRs (i.e., rectangular GNFs) [5], hexagon-shaped GNFs [12], and triangle-shaped GNFs [15] in recent years. According to the findings, the electronic properties associated with these GNFs are very different. Therefore, here we continue employing TAO-DFT to explore the electronic properties associated with *n*-pyrenes with *n* = 2–15 (i.e., diamond-shaped GNFs of different sizes), which could pave the way for their potential applications in electronics and optoelectronics.

## 2. Computational Details

Geometry optimizations and single-point energy calculations are all performed using TAO-LDA [40], which is TAO-DFT employing the LDA θ-dependent energy functional and XC energy functional, where the recommended value of θ = 7 × 10${}^{-3}$ (hartree) is employed [40]. All computational results are obtained with Q-Chem 4.4 [55], adopting the 6-31G(d) basis set (i.e., a valence double-zeta polarized basis set) as well as a numerical quadrature which contains 75 grid points in the Euler-Maclaurin radial grid and 302 grid points in the Lebedev angular grid.

All calculations are performed under isolated boundary conditions (i.e., well suited for studying molecules). A convergence tolerance of 10${}^{-8}$ (hartree) is set for single-point energy calculations. For the convergence criteria of geometry optimizations, we adopt the default tolerances of Q-Chem (i.e., geometry optimizations are considered converged, when a convergence tolerance of 3 × 10${}^{-4}$ (hartree/bohr) on the maximum gradient component is reached, and either a convergence tolerance of 1.2 × 10${}^{-3}$ (bohr) on the maximum atomic displacement or a convergence tolerance of 10${}^{-6}$ (hartree) on the energy change of successive optimization cycles is reached).

## 3. Results and Discussion

### 3.1. Singlet-Triplet Energy Gap

To determine the ground state, we first optimize the structures of *n*-pyrene associated with the lowest singlet state and lowest triplet state, by spin-unrestricted TAO-LDA, and thereafter calculate the difference between the lowest singlet energy ($E_{\mathrm{singlet}}$) and lowest triplet energy ($E_{\mathrm{triplet}}$) associated with *n*-pyrene using [5,12,15,40,41,42,45,46,47,48,49,50,51]

(1) $$E_{\mathrm{ST}}=E_{\mathrm{triplet}}-E_{\mathrm{singlet}},$$

where $E_{\mathrm{ST}}$ is the singlet-triplet energy gap of *n*-pyrene.

**Figure 2:**
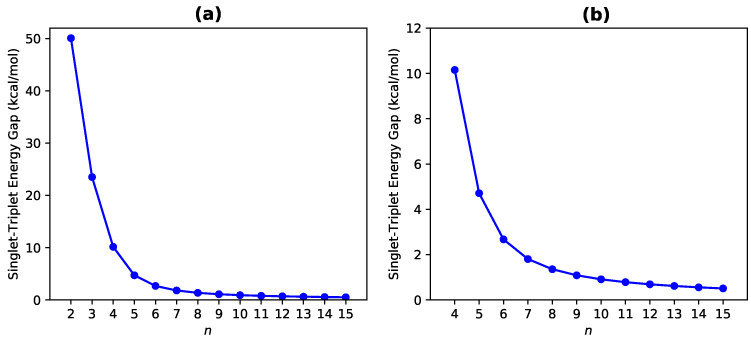
Singlet-triplet energy gap of *n*-pyrene ((**a**) *n* = 2–15 and (**b**) *n* = 4–15), calculated by spin-unrestricted TAO-LDA.

As can be seen in Figure 2, the $E_{\mathrm{ST}}$ value of *n*-pyrene remains positive for each *n* value (also see Table S1 in Supplementary Information (SI)). Therefore, all the *n*-pyrenes studied are ground-state singlets. As the size of *n*-pyrene increases, the $E_{\mathrm{ST}}$ value monotonically decreases at a faster rate for smaller *n* = 2–4, followed by a slower rate of monotonic decrease for larger values of *n*. Understanding the $E_{\mathrm{ST}}$ values is crucially important for certain applications, such as the thermal energy conversion where the singlet-fission phenomenon is utilized [56]. Besides, exploring systems with extremely small (or even vanishing) values of $E_{\mathrm{ST}}$ is also demanding for the thermally activated delayed fluorescence (TADF) applications [57,58,59]. On the basis of the $E_{\mathrm{ST}}$ values reported, the larger *n*-pyrenes can be useful for the singlet-fission phenomenon and TADF applications.

KS-DFT employing the widely adopted XC energy functionals can suffer from the unphysical symmetry-breaking problems in the respective spin-unrestricted calculations [31,32], especially for systems with radical character. For an exact theory (where there is no unphysical symmetry-breaking problem) [36,40,60], the spin-unrestricted and spin-restricted calculations must yield identical energy. To investigate whether spin-unrestricted TAO-LDA resolves unphysical symmetry-breaking problems, we additionally perform geometry optimizations for the lowest singlet states of *n*-pyrenes by spin-restricted TAO-LDA. The requirement of symmetry can indeed be satisfied for *n* = 2–15 (i.e., for all the *n* values considered), as the TAO-LDA spin-unrestricted and spin-restricted singlet energies of *n*-pyrenes are essentially the same.

### 3.2. Vertical Ionization Potential/Electron Affinity as Well as Fundamental Gap

Now, we explore if *n*-pyrenes (containing *N* electrons) could be useful for applications in optoelectronics. At the ground-state geometry, we perform spin-unrestricted TAO-LDA calculations to compute the vertical ionization potential of ground-state *n*-pyrene using [5,12,15,41,42,46,47,48,49,50,51]

(2) $${\mathrm{IP}}_{v}=E_{N-1}-E_{N},$$

the vertical electron affinity of ground-state *n*-pyrene using

(3) $${\mathrm{EA}}_{v}=E_{N}-E_{N+1},$$

and the fundamental gap (i.e., ${\mathrm{IP}}_{v}-{\mathrm{EA}}_{v}$) of ground-state *n*-pyrene using

(4) $$E_{g}=E_{N+1}+E_{N-1}-2E_{N},$$

where $E_{N}$, $E_{N-1}$, and $E_{N+1}$ are the energies associated with neutral *n*-pyrene, cationic *n*-pyrene, and anionic *n*-pyrene, respectively.

**Figure 3:**
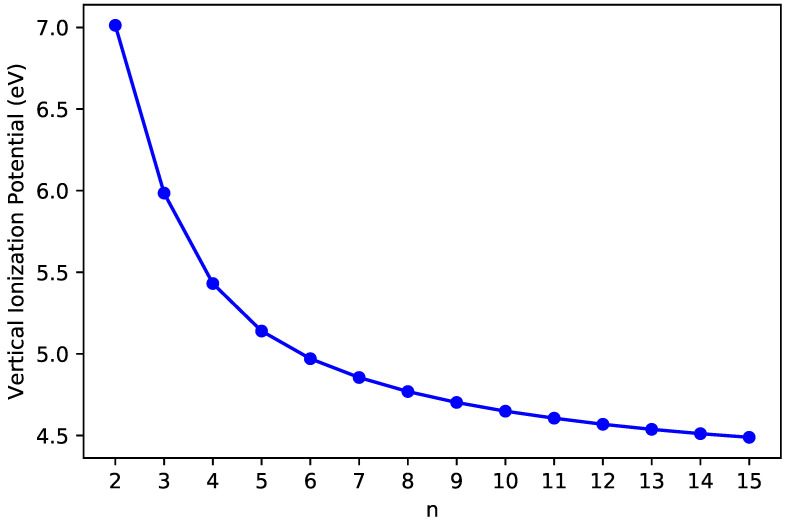
Vertical ionization potential of ground-state *n*-pyrene, calculated by spin-unrestricted TAO-LDA.

**Figure 4:**
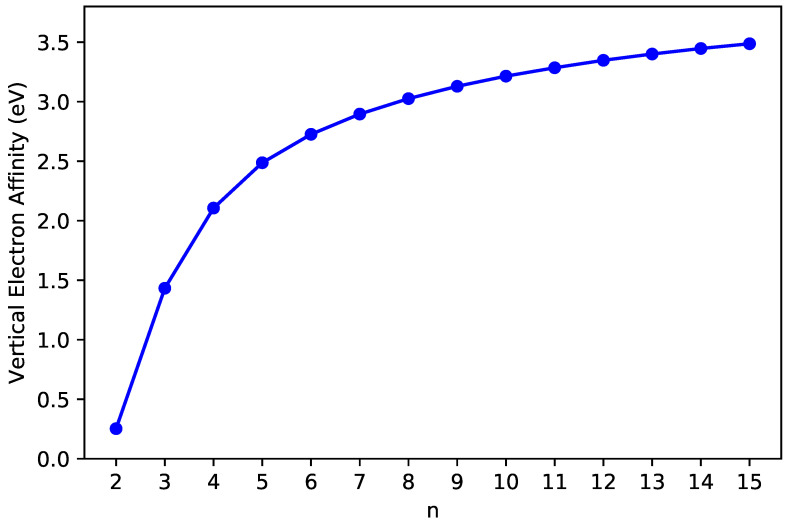
Vertical electron affinity of ground-state *n*-pyrene, calculated by spin-unrestricted TAO-LDA.

**Figure 5:**
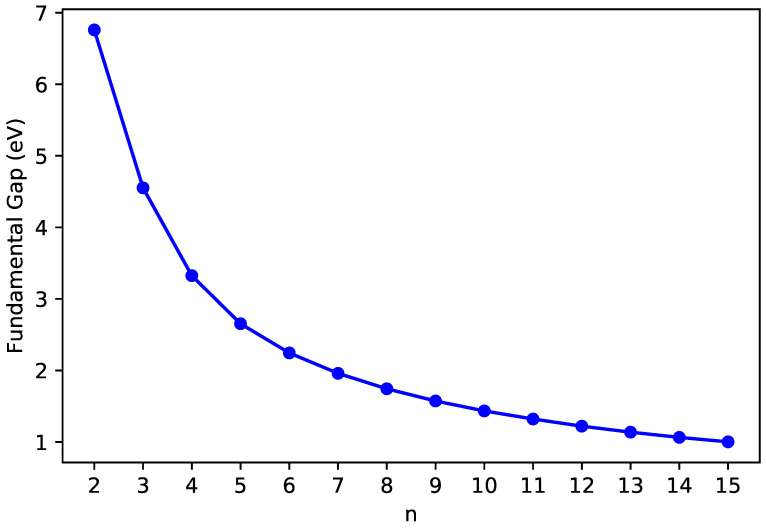
Fundamental gap of ground-state *n*-pyrene, calculated by spin-unrestricted TAO-LDA.

The ${\mathrm{IP}}_{v}$ (Figure 3), ${\mathrm{EA}}_{v}$ (Figure 4), and $E_{g}$ (Figure 5) values of ground-state *n*-pyrene are presented as functions of *n* (also see Table S2 in SI). With the increase of the size of *n*-pyrene, the ${\mathrm{IP}}_{v}$ value decreases monotonically, and the ${\mathrm{EA}}_{v}$ value increases monotonically, which results in a monotonic decrease in the $E_{g}$ value (i.e., the difference between the ${\mathrm{IP}}_{v}$ value and the ${\mathrm{EA}}_{v}$ value). Interestingly, *n*-pyrenes (with *n* = 5–15) possess the $E_{g}$ values lying between 1 eV and 3 eV, showing promise for their applications in nanophotonics.

### 3.3. Symmetrized von Neumann Entropy

For a quantitative measure of the radical character of ground-state *n*-pyrene, we compute the symmetrized von Neumann entropy [5,12,15,41,42,46,47,48,49,50,51,60]

(5) $$S_{\mathrm{vN}}=-\frac{1}{2}\sum_{\sigma =↑,↓}\sum_{i=1}^{\infty }\left\{f_{i,\sigma }\;\ln\left(f_{i,\sigma }\right)+\left(1-f_{i,\sigma }\right)\;\ln\left(1-f_{i,\sigma }\right)\right\},$$

by spin-unrestricted TAO-LDA. Note that $f_{i,\sigma }$, which ranges from zero to one, is the occupation number of the $i^{\mathrm{th}}$σ-spin (i.e., up-spin or down-spin) orbital from spin-unrestricted TAO-LDA, and is closely related to the occupation number of the $i^{\mathrm{th}}$ natural orbital of σ-spin [40,41,42]. For an electronic system with nonradical character, the occupation numbers of all spin-orbitals are in the vicinity of either 0 or 1, making only a vanishingly small contribution to the respective value of $S_{\mathrm{vN}}$. Nevertheless, for an electronic system possessing pronounced radical character, the occupation numbers of active spin-orbitals (which are the spin-orbitals with significant fractional occupations) can be rather different from 0 and 1 (e.g., in the range of 0.1 to 0.9); hence, the respective value of $S_{\mathrm{vN}}$ can be greatly increased when the number of active spin-orbitals increases and/or the occupation numbers associated with active spin-orbitals become closer to 0.5.

**Figure 6:**
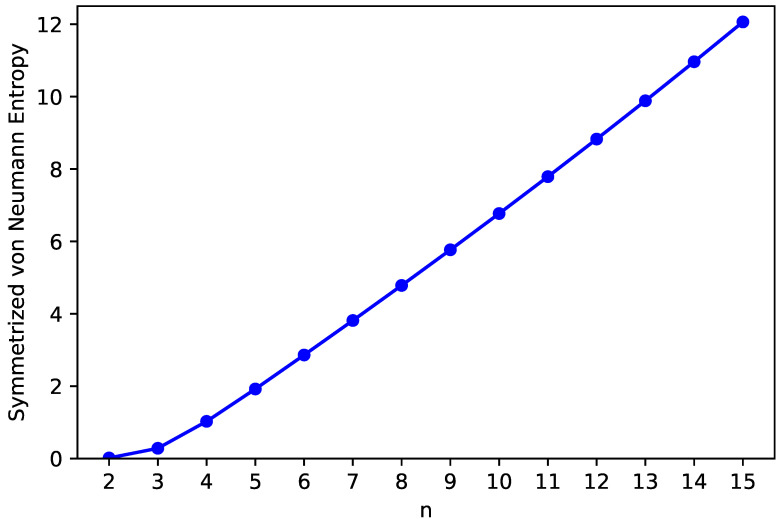
Symmetrized von Neumann entropy of ground-state *n*-pyrene, calculated by spin-unrestricted TAO-LDA.

As presented in Figure 6, the $S_{\mathrm{vN}}$ value of ground-state *n*-pyrene increases monotonically with increasing system size, which implies that the larger ground-state *n*-pyrenes could exhibit increasing polyradical nature (also see Table S2 in SI).

### 3.4. Active Orbital Occupation Numbers

**Figure 7:**
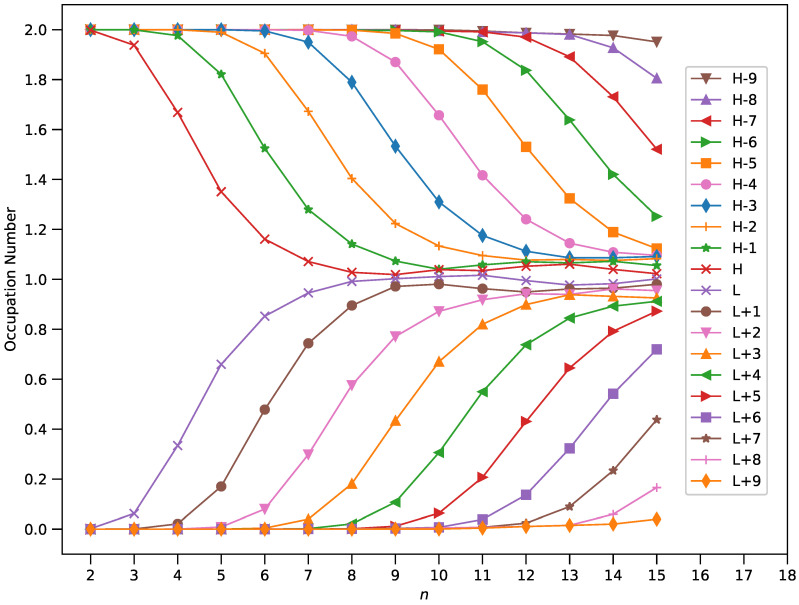
Active orbital occupation numbers (H−9, H−8, …, H, L, …, L+8, and L+9) of ground-state *n*-pyrene, calculated by spin-restricted TAO-LDA. HOMO/LUMO is denoted as H/L for brevity.

In order to unlock the mystery of increasing $S_{\mathrm{vN}}$ value with the size of *n*-pyrene, we present the active orbital occupation numbers of ground-state *n*-pyrene (with *N* electrons), computed using spin-restricted TAO-LDA (see Figure 7). Here, we define the HOMO (i.e., highest occupied molecular orbital) as the ${\left(N/2\right)}^{\mathrm{th}}$ orbital, the LUMO (i.e., lowest unoccupied molecular orbital) as the ${\left(N/2+1\right)}^{\mathrm{th}}$ orbital, and so on [5,12,15,40,42,45,47,50,51]. Besides, the active orbitals are regarded as the orbitals possessing an occupation number in the range of 0.2 to 1.8.

For smaller values of *n* (up to *n* = 3), the occupation numbers of all orbitals are in the vicinity of either 0 or 2. Accordingly, 2-pyrene and 3-pyrene are expected to possess nonradical character. However, when *n* increases, the number of active orbitals increases and/or the occupation numbers associated with active orbitals become closer to 1, indicating that the larger ground-state *n*-pyrenes could exhibit increasing polyradical nature (also see Table S3 in SI).

From the occupation number analysis, the smaller ground-state *n*-pyrenes (e.g., n≤3) exhibit nonradical nature, which is consistent with the analyses of the other electronic properties of these stable *n*-pyrenes (e.g., the larger values of $E_{\mathrm{ST}}$, the larger values of $E_{g}$, and the smaller values of $S_{\mathrm{vN}}$). On the other hand, the larger ground-state *n*-pyrenes (e.g., n>3) exhibit increasing polyradical nature, which is also consistent with the analyses of the other electronic properties of these relatively unstable *n*-pyrenes (e.g., the smaller values of $E_{\mathrm{ST}}$, the smaller values of $E_{g}$, and the larger values of $S_{\mathrm{vN}}$).

### 3.5. Real-Space Representation of Active Orbitals

**Figure 8:**
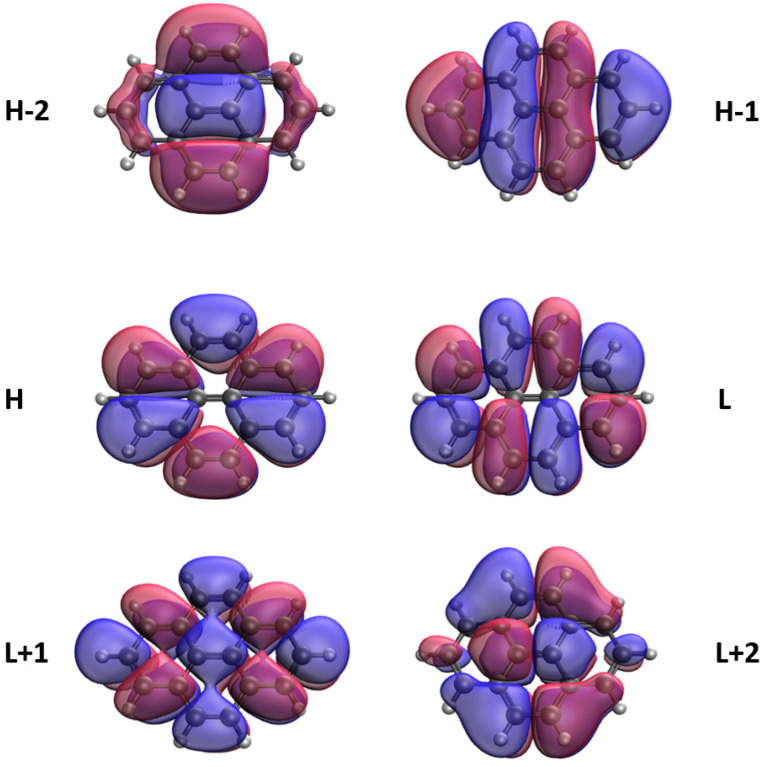
Real-space representation of H−2 (2.000), H−1 (2.000), H (1.998), L (0.002), L+1 (0.000), and L+2 (0.000) of ground-state 2-pyrene, calculated by spin-restricted TAO-LDA. Here the isovalue is 0.02 e/Å${}^{3}$. The occupation numbers of active orbitals are given in parentheses, and HOMO/LUMO is denoted as H/L for brevity.

**Figure 9:**
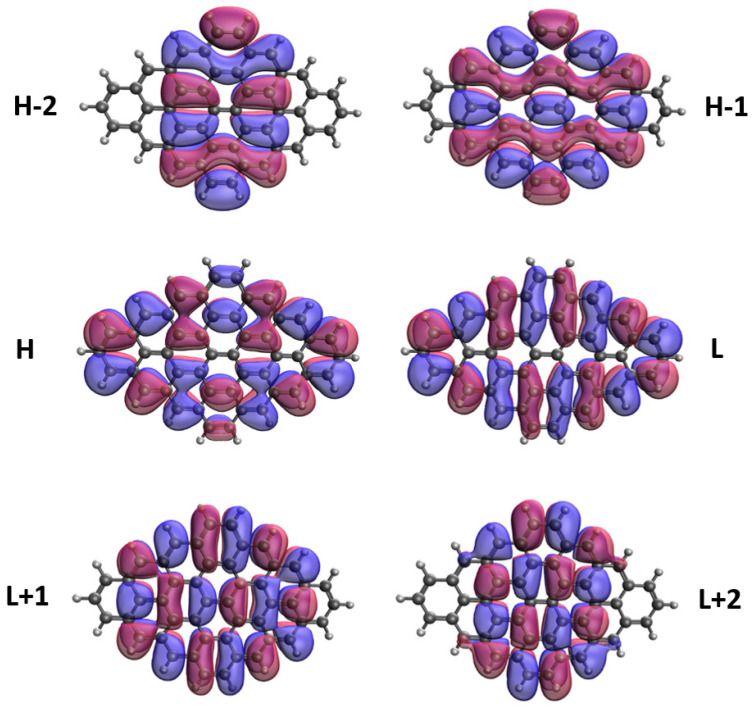
Real-space representation of H−2 (2.000), H−1 (1.976), H (1.669), L (0.334), L+1 (0.021), and L+2 (0.000) of ground-state 4-pyrene, calculated by spin-restricted TAO-LDA. Here the isovalue is 0.02 e/Å${}^{3}$. The occupation numbers of active orbitals are given in parentheses, and HOMO/LUMO is denoted as H/L for brevity.

**Figure 10:**
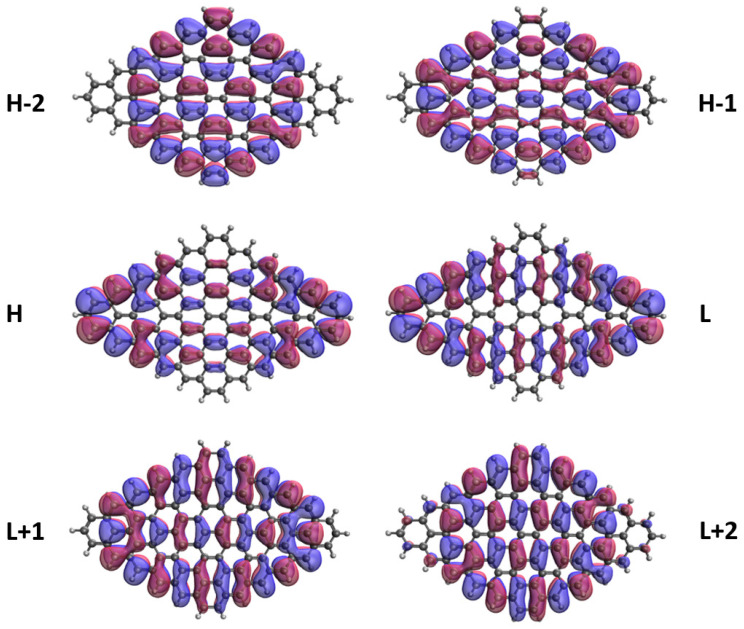
Real-space representation of H−2 (1.905), H−1 (1.524), H (1.161), L (0.852), L+1 (0.478), and L+2 (0.080) of ground-state 6-pyrene, calculated by spin-restricted TAO-LDA. Here the isovalue is 0.02 e/Å${}^{3}$. The occupation numbers of active orbitals are given in parentheses, and HOMO/LUMO is denoted as H/L for brevity.

**Figure 11:**
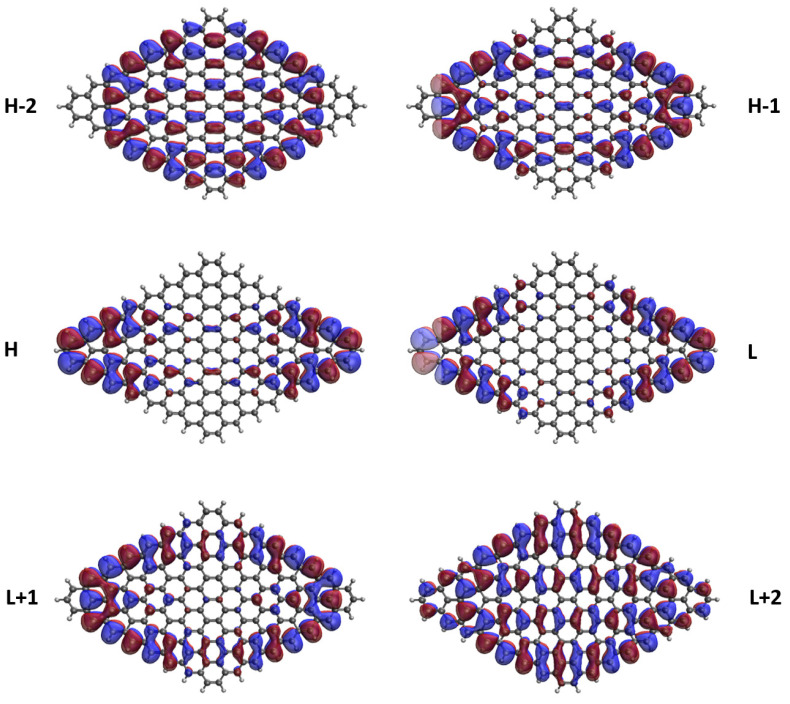
Real-space representation of H−2 (1.404), H−1 (1.141), H (1.028), L (0.992), L+1 (0.895), and L+2 (0.576) of ground-state 8-pyrene, calculated by spin-restricted TAO-LDA. Here the isovalue is 0.02 e/Å${}^{3}$. The occupation numbers of active orbitals are given in parentheses, and HOMO/LUMO is denoted as H/L for brevity.

**Figure 12:**
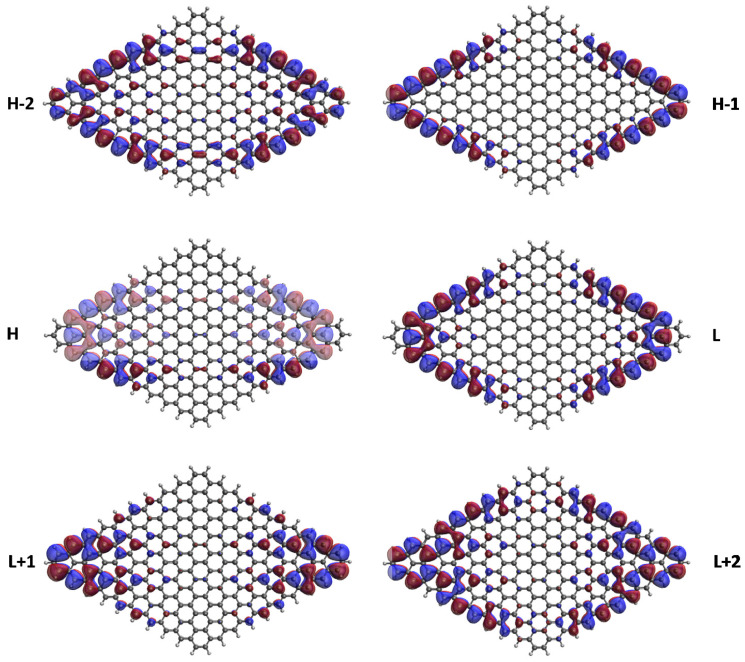
Real-space representation of H−2 (1.133), H−1 (1.040), H (1.038), L (1.011), L+1 (0.981), and L+2 (0.872) of ground-state 10-pyrene, calculated by spin-restricted TAO-LDA. Here the isovalue is 0.02 e/Å${}^{3}$. The occupation numbers of active orbitals are given in parentheses, and HOMO/LUMO is denoted as H/L for brevity.

We explore the real-space representation of active orbitals for the ground states of some representative *n*-pyrenes, such as 2-pyrene (see Figure 8), 4-pyrene (see Figure 9), 6-pyrene (see Figure 10), 8-pyrene (see Figure 11), and 10-pyrene (see Figure 12), computed using spin-restricted TAO-LDA. As shown, the active orbitals of the smaller *n*-pyrenes (e.g., n≤3) appear delocalized over the whole *n*-pyrenes. Nonetheless, when *n* increases, more and more active orbitals become concentrated on the zigzag edges of the larger *n*-pyrenes, which is expected to be intimately related to the increasing polyradical nature of the larger *n*-pyrenes. This implies that when available, these edge states should be energetically more preferred than other states. Since more edge states become available for the larger *n*-pyrenes, more unpaired electrons become concentrated on the zigzag edges of the larger *n*-pyrenes. Nevertheless, the reason of this correlation (i.e., the connection between edge states and radical formation) remains unclear, and hence, it can be of great interest to develop a simple model to qualitatively explain this correlation in the future.

## 4. Conclusions

In conclusion, the radical character associated with GNFs has posed a serious challenge to common computational approaches. The challenge has been greatly resolved by the recently formulated TAO-DFT, which is a highly efficient computational method for exploring the electronic properties associated with nanosystems exhibiting radical nature. Here, we have adopted TAO-DFT to explore the electronic properties associated with *n*-pyrenes with *n* = 2–15. The larger ground-state *n*-pyrenes have been found to be polyradicals, playing a significant role in the determination of their electronic properties. Because of the polyradical character associated with the larger *n*-pyrenes, studying their electronic properties using KS-DFT with the commonly employed XC energy functionals may not be reliable, and studying their electronic properties using accurate MR electronic structure methods may not be computationally feasible. Accordingly, studying the electronic properties of *n*-pyrenes using TAO-DFT in the present work is definitely justified.

From our TAO-DFT findings, *n*-pyrenes are ground-state singlets for *n* = 2–15 (i.e., for all the *n* values considered). With increasing the size of *n*-pyrene, the value of $E_{\mathrm{ST}}$, the value of ${\mathrm{IP}}_{v}$, and the value of $E_{g}$ monotonically decrease, while the value of ${\mathrm{EA}}_{v}$ and the value of $S_{\mathrm{vN}}$ monotonically increase. When *n* increases, there is a smooth transition from the nonradical nature of the smaller *n*-pyrenes (e.g., n≤3) to the increasing polyradical nature of the larger *n*-pyrenes (e.g., n>3). Furthermore, the latter is expected to be intimately correlated with the increasing concentration of active orbitals on the zigzag edges of the larger *n*-pyrenes. 

## Supplementary Materials

The following are available online at https://www.mdpi.com/2079-4991/10/6/1236/s1, Table S1: Singlet-triplet energy gap $E_{\mathrm{ST}}$ (in kcal/mol) of *n*-pyrene, calculated by spin-unrestricted TAO-LDA, Table S2: Vertical ionization potential ${\mathrm{IP}}_{v}$ (in eV), vertical electron affinity ${\mathrm{EA}}_{v}$ (in eV), fundamental gap $E_{g}$ (in eV), and symmetrized von Neumann entropy $S_{\mathrm{vN}}$ of ground-state *n*-pyrene, calculated by spin-unrestricted TAO-LDA, Table S3: Active orbital occupation numbers (HOMO−9, HOMO−8, …, HOMO, LUMO, …, LUMO+8, and LUMO+9) of ground-state *n*-pyrene, calculated by spin-restricted TAO-LDA. For brevity, HOMO/LUMO is denoted as H/L.
