# Supplementary material for: TAO-DFT Study on the Electronic Properties of Diamond-Shaped Graphene Nanoflakes
Source: Nanomaterials (Basel). 2020 Jun 25;10(6):1236. doi: 10.3390/nano10061236 (PMC7353095; doi:10.3390/nano10061236)
Supplement: Supplementary file 1 [file nanomaterials-10-01236-s001.pdf]

Supplementary Information to: TAO-DFT Study on the Electronic Properties of Diamond-Shaped Graphene Nanoflakes

Hong-Jui Huang,[1,*] Sonai Seenithurai,[1,*] and Jeng-Da Chai[1,2,†]

[1] *Department of Physics, National Taiwan University, Taipei 10617, Taiwan*

[2] *Center for Theoretical Physics and Center for Quantum Science and Engineering, National Taiwan University, Taipei 10617, Taiwan*

[*] These authors contributed equally to this work.

[†] Corresponding author. E-mail: jdchai@phys.ntu.edu.tw

LIST OF TABLES

S1 Singlet-triplet energy gap E_{ST} (in kcal/mol) of n-pyrene, calculated by spin-unrestricted TAO-LDA. 3

S2 Vertical ionization potential IP_v (in eV), vertical electron affinity EA_v (in eV), fundamental gap E_g (in eV), and symmetrized von Neumann entropy S_{vN} of ground-state n-pyrene, calculated by spin-unrestricted TAO-LDA. 3

S3 Active orbital occupation numbers (HOMO−9, HOMO−8, ..., HOMO, LUMO, ..., LUMO+8, and LUMO+9) of ground-state n-pyrene, calculated by spin-restricted TAO-LDA. For brevity, HOMO/LUMO is denoted as H/L. 4

TABLES

TABLE S1. Singlet-triplet energy gap $E_{\rm ST}$ (in kcal/mol) of n-pyrene, calculated by spin-unrestricted TAO-LDA.

n	$E_{\rm ST}$
2	50.07
3	23.50
4	10.15
5	4.71
6	2.67
7	1.80
8	1.36
9	1.09
10	0.91
11	0.78
12	0.69
13	0.62
14	0.55
15	0.51

TABLE S2. Vertical ionization potential IP_v (in eV), vertical electron affinity EA_v (in eV), fundamental gap E_g (in eV), and symmetrized von Neumann entropy $S_{\rm vN}$ of ground-state n-pyrene, calculated by spin-unrestricted TAO-LDA.

n	IP_v	EA_v	E_g	$S_{\rm vN}$
2	7.01	0.25	6.76	0.02
3	5.99	1.43	4.55	0.28
4	5.43	2.11	3.32	1.03
5	5.14	2.49	2.65	1.92
6	4.97	2.73	2.24	2.86

7	4.86	2.90	1.96	3.81
8	4.77	3.03	1.74	4.78
9	4.70	3.13	1.57	5.77
10	4.65	3.21	1.43	6.77
11	4.61	3.29	1.32	7.79
12	4.57	3.35	1.22	8.83
13	4.54	3.40	1.14	9.88
14	4.51	3.45	1.06	10.96
15	4.49	3.49	1.00	12.06

TABLE S3. Active orbital occupation numbers (HOMO−9, HOMO−8, ..., HOMO, LUMO, ..., LUMO+8, and LUMO+9) of ground-state n-pyrene, calculated by spin-restricted TAO-LDA. For brevity, HOMO/LUMO is denoted as H/L.

n	2	3	4	5	6	7	8	9	10	11	12	13	14	15
H−9	2.000	2.000	2.000	2.000	2.000	2.000	2.000	2.000	1.999	1.995	1.987	1.982	1.977	1.951
H−8	2.000	2.000	2.000	2.000	2.000	2.000	2.000	1.999	1.994	1.991	1.987	1.981	1.927	1.805
H−7	2.000	2.000	2.000	2.000	2.000	2.000	1.998	1.997	1.994	1.991	1.970	1.891	1.731	1.521
H−6	2.000	2.000	2.000	2.000	2.000	1.999	1.998	1.997	1.991	1.952	1.837	1.638	1.420	1.252
H−5	2.000	2.000	2.000	2.000	2.000	1.999	1.998	1.985	1.921	1.760	1.530	1.324	1.189	1.123
H−4	2.000	2.000	2.000	2.000	2.000	1.997	1.973	1.870	1.657	1.417	1.241	1.145	1.108	1.096
H−3	2.000	2.000	2.000	2.000	1.995	1.950	1.789	1.533	1.310	1.176	1.112	1.086	1.086	1.091
H−2	2.000	2.000	2.000	1.990	1.905	1.673	1.404	1.223	1.133	1.095	1.077	1.080	1.075	1.083
H−1	2.000	1.999	1.976	1.821	1.524	1.280	1.141	1.073	1.040	1.058	1.071	1.066	1.072	1.055
H	1.998	1.938	1.669	1.351	1.161	1.071	1.028	1.019	1.038	1.035	1.052	1.061	1.040	1.022
L	0.002	0.062	0.334	0.660	0.852	0.946	0.992	1.002	1.011	1.016	0.995	0.977	0.982	1.001
L+1	0.000	0.001	0.021	0.170	0.478	0.744	0.895	0.971	0.981	0.963	0.949	0.961	0.964	0.980
L+2	0.000	0.000	0.000	0.008	0.080	0.299	0.576	0.771	0.872	0.918	0.942	0.940	0.961	0.955
L+3	0.000	0.000	0.000	0.000	0.004	0.040	0.181	0.434	0.670	0.820	0.899	0.938	0.932	0.925
L+4	0.000	0.000	0.000	0.000	0.000	0.002	0.021	0.107	0.306	0.550	0.738	0.845	0.892	0.912
L+5	0.000	0.000	0.000	0.000	0.000	0.001	0.001	0.012	0.064	0.207	0.431	0.645	0.792	0.872

L+6	0.000	0.000	0.000	0.000	0.000	0.001	0.001	0.003	0.007	0.038	0.137	0.323	0.542	0.719
L+7	0.000	0.000	0.000	0.000	0.000	0.000	0.001	0.003	0.005	0.007	0.024	0.090	0.234	0.438
L+8	0.000	0.000	0.000	0.000	0.000	0.000	0.000	0.001	0.005	0.007	0.011	0.015	0.059	0.166
L+9	0.000	0.000	0.000	0.000	0.000	0.000	0.000	0.000	0.001	0.004	0.011	0.015	0.020	0.040
